# A Mobile Cough Strength Evaluation Device Using Cough Sounds

**DOI:** 10.3390/s18113810

**Published:** 2018-11-07

**Authors:** Yasutaka Umayahara, Zu Soh, Kiyokazu Sekikawa, Toshihiro Kawae, Akira Otsuka, Toshio Tsuji

**Affiliations:** 1 Department of System Cybernetics, Institute of Engineering, Hiroshima University, 1-4-1 Kagamiyama, Higashi-Hiroshima, Hiroshima 739-8527, Japan; sozu@bsys.hiroshima-u.ac.jp; 2 Department of Rehabilitation, Faculty of Health Sciences, Hiroshima Cosmopolitan University, Hiroshima 731-3166, Japan; otsuka-1949@hcu.ac.jp; 3 Division of Physical Analysis and Therapeutic Sciences, Institute of Biomedical and Health Sciences, Hiroshima University, Hiroshima 734-8553, Japan; sekikawa@hiroshima-u.ac.jp; 4 Division of Rehabilitation, Department of Clinical Support, Hiroshima University Hospital, Hiroshima 734-8551, Japan; toshihiro@hiroshima-u.ac.jp

**Keywords:** cough strength, cough sound, cough peak flow, mobile device, iOS

## Abstract

Although cough peak flow (CPF) is an important measurement for evaluating the risk of cough dysfunction, some patients cannot use conventional measurement instruments, such as spirometers, because of the configurational burden of the instruments. Therefore, we previously developed a cough strength estimation method using cough sounds based on a simple acoustic and aerodynamic model. However, the previous model did not consider age or have a user interface for practical application. This study clarifies the cough strength prediction accuracy using an improved model in young and elderly participants. Additionally, a user interface for mobile devices was developed to record cough sounds and estimate cough strength using the proposed method. We then performed experiments on 33 young participants (21.3 ± 0.4 years) and 25 elderly participants (80.4 ± 6.1 years) to test the effect of age on the CPF estimation accuracy. The percentage error between the measured and estimated CPFs was approximately 6.19%. In addition, among the elderly participants, the current model improved the estimation accuracy of the previous model by a percentage error of approximately 6.5% (*p* < 0.001). Furthermore, Bland-Altman analysis demonstrated no systematic error between the measured and estimated CPFs. These results suggest that the developed device can be applied for daily CPF measurements in clinical practice.

## 1. Introduction

The cough is an important defence mechanism for clearing excess secretions and foreign materials from airways [1,2]. Generally, cough peak flows (CPFs) are measured using a spirometer or a peak flow meter to assess cough strength because cough strength reflects the ability to clear secretions from the respiratory tract and indicates the aspiration risk. For example, it is unlikely that someone with a CPF greater than 270 L/min will develop acute respiratory distress [3]. Patients with a CPF greater than 160 L/min can manage ventilatory failure without a tracheostomy [4,5]. Dysphagic patients with persistent tracheobronchial aspiration with a CPF less than 242 L/min have a high risk of developing pulmonary complications [6]. Although previous studies have reported the importance of CPF as a measurement for assessing the ability to expel airway secretions, it requires a face mask and a bacterial filter firmly attached to the patient’s mouth, as well as configuration of a complex measurement instrument. This method imposes burdens on both patients and their caregivers, and prevents daily CPF measurements in clinical practice.

To solve this problem, our research group developed a CPF estimation method using cough sounds [7,8] by modelling the relationship between the cough sounds and flow rates. The method enabled the estimation of CPF without a face mask or bacterial filter, and drastically simplified the measurement instrument configuration because the cough sounds can easily be measured using a microphone. However, the previous method did not fully consider the effect of age, body weight, or body mass index (BMI), although relationships between CPF and age and body weight have been reported [9]. In addition, the previous method could not immediately present the estimated result to the patient, as it lacked a user interface. These limitations made it difficult to apply the proposed method in clinical practice.

Therefore, this paper presents a newly proposed CPF estimation model that can account for the relationship between CPFs and cough sounds while considering age, body weight, and BMI. In addition, aiming for future applications in the clinic and the home, we also present a user interface incorporating the proposed CPF estimation model for mobile devices that enables cough sound recording, immediate CPF estimation, and estimated CPF history management. Finally, we report the estimation accuracy of the proposed model implemented using a mobile device by performing experiments on young participants (n = 33) and elderly participants (n = 25).

## 2. Proposed Device

In this paper, we propose a CPF estimation model and user interface software for iOS version 11.4 to assess cough strength. The details of the proposed model and the user interface are described in this section.

### 2.1. Model

The proposed model is expressed by the following equation:

(1) $$CPS_{proposed}=\left(\alpha_{0,\left(1\right)}+\alpha_{1,\left(1\right)}\cdot age\right)\cdot \left(exp^{\beta_{\left(1\right)}\cdot CPSL}-1\right),$$

where *α*_0,(1)_, *α*_1,(1)_ and *β*_(1)_ are the parameters predetermined by a non-linear regression analysis (the Levenberg-Marquardt method) using the mean square error between the *CPS_proposed_* and the measured CPF as the evaluation function. The *CPSL* represents the cough peak sound pressure level. This model indicates that the cough peak flow (estimated CPF (*CPS_proposed_*)) and the *CPSL* are logarithmically related. The next section describes the preprocessing method used to derive the *CPSL* from cough sounds, which can be measured using a smartphone.

### 2.2. Measurement Protocol and Preprocessing for Cough Sounds

**Figure 1:**
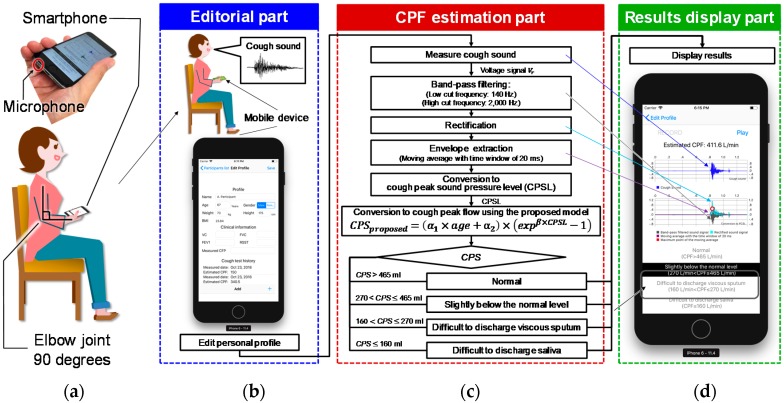
Cough sound measurement protocol and software configuration. *CPS* represents the cough peak flow estimated using cough sounds. (**a**) The posture of a user while recording cough sounds. (**b**) The user interface for editing a personal profile. (**c**) The flow chart for cough sound preprocessing and cough peak flow (CPF) estimation. (**d**) The results display that demonstrates the graphs resulting from signal preprocessing and CPF estimation.

Cough sounds are measured using the microphone built into a smartphone (iPhone 6 A1586; Apple, Inc., Cupertino, CA, USA), which is hereafter referred to as the smartphone microphone. While measuring cough sounds, the user was required to hold the bottom of the smartphone in their right hand with the elbow flexed to 90°, the shoulder at 0°, and the elbow internally rotated to 45° (Figure 1a,b). Figure 1a shows how to hold the smartphone and the location of the microphone. The smartphone screen was directed towards the participant’s mouth so that the microphone was directed to the body of the user to avoid recording the sound of exhaled air. Figure 1c shows the flowchart for measuring and analysing cough sounds. The cough sound signals are digitized by the smartphone’s built-in 16-bit AD converter at a sampling rate of 48 kHz and saved in the native.wav format on the device. The recorded cough sound is then preprocessed as shown in the flowchart in Figure 1c. The digitized cough sound signals are bandpass-filtered between 140 and 2000 Hz to minimize artefacts caused by heart sounds and muscle interference. Subsequently, the cough sound signals are rectified by calculating their absolute values and smoothed using a 20-ms time window to extract the envelope [10], which represents the cough sound pressure level. The value of the *CPSL* is then calculated from the maximal value of the envelope. The *CPSL* is then substituted into the proposed model, shown in Equation (1), to estimate the cough peak flow (*CPS_proposed_*).

### 2.3. User Interface Software

The proposed user interface is composed of three parts: The editorial part, the CPF estimation part, and the results display part. The interface starts from the editorial part, which prompts the user to complete their personal profile and proceed to record cough sounds. The CPF estimation part estimates the CPF from the recorded cough sounds using the proposed model. Finally, the estimated CPF is shown in the results display part. Each part of the interface is described below.

**Figure 2:**
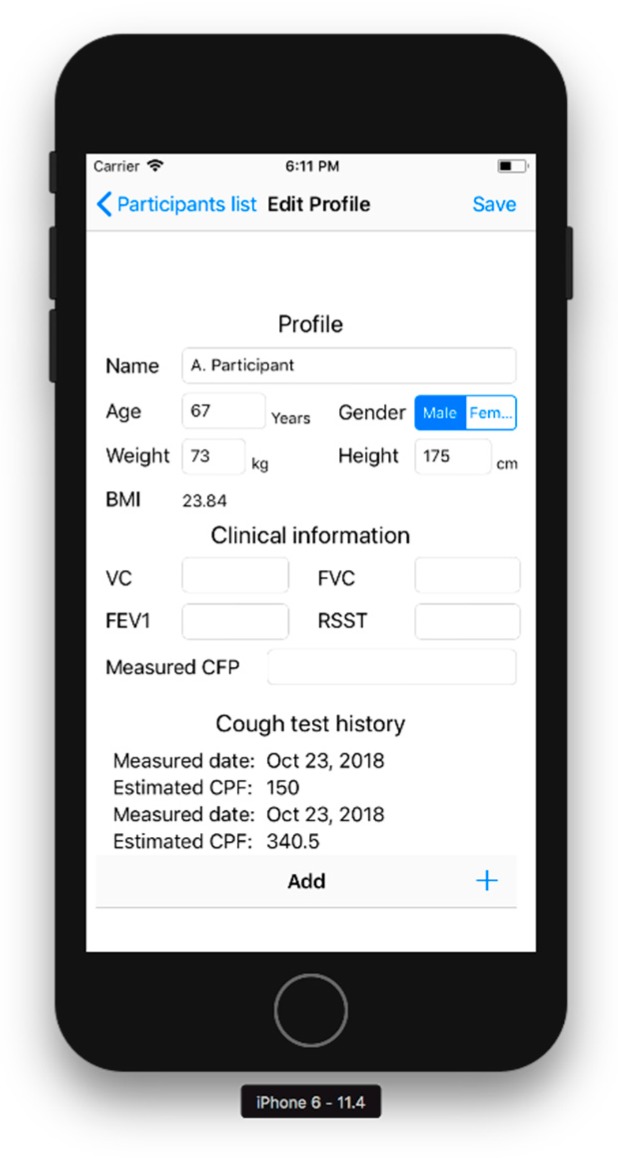
Editorial part. This screen prompts the user to complete their personal profile. BMI, body mass index, BMI = body weight/height^2^; VC, vital capacity; FVC, forced vital capacity; FEV_1_, forced expiratory volume in 1 s; RSST, repetitive saliva swallowing test.

Editorial part: Figure 2 shows the editorial part. As the initial collection of the participant’s characteristics, this part prompts the user to input their name, age, sex, height, and weight. BMI is automatically calculated from the height and weight. The editorial part can also record respiratory functions, such as the vital capacity (VC), forced vital capacity (FVC), forced expiratory volume in one second (FEV_1_), measured CPF, and results of an objective swallowing function test, such as the repetitive saliva swallowing test (RSST), which detects patients who experience aspiration [10,11,12]. The bottom of the screen shows the history of the estimated CPFs to inform the user of chronical changes in cough function.

**Figure 3:**
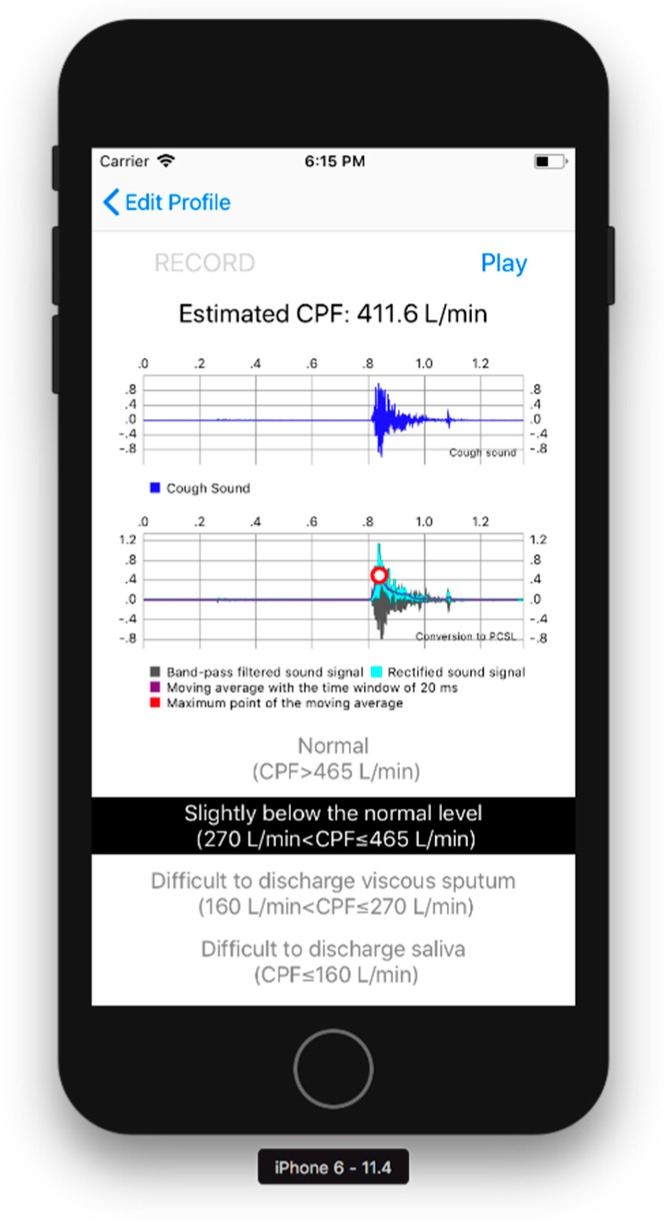
Results display part. The blue solid line represents the measured cough sound signal. The light blue solid line represents the rectified sound signal. The purple solid line represents the moving average with a time window of 20 ms. The red dot represents the maximum point of the moving average.

CPF estimation part: The CPF estimation part records the cough sounds, converting the cough sounds into the *CPSL*, and estimates the CPF as described in Section 2.1 and Section 2.2. A user can record cough sounds by pressing the “RECORD” button, as shown in Figure 3. The *CPS* is then calculated when the “play” button at the upper right of the screen is pressed, as shown in Figure 3.

Results display part: Figure 3 shows the results display part. The waveform of the cough sounds, the rectified signal, the envelope, and the maximum value of the envelope can be confirmed from the graphs shown in the centre of the screen. The estimated cough peak flow (*CPS_proposed_*) is classified into four risk levels based on previously reported cut-off values [3,4,5,11], as shown below and in Figure 3:

$$\begin{array}{cc} & CPS_{proposed}>465\;L/\min:\;\mathrm{Normal}\\ 270\;L/\min< & CPS_{proposed}\leq 465\;L/\min:\;\mathrm{Slightly}\;\mathrm{below}\;\mathrm{the}\;\mathrm{normal}\;\mathrm{level}\\ 160\;L/\min< & CPS_{proposed}\leq 270\;L/\min:\;\mathrm{Difficult}\;\mathrm{to}\;\mathrm{discharge}\;\mathrm{viscous}\;\mathrm{sputum}\\ & CPS_{proposed}\leq 160\;L/\min:\;\mathrm{Difficult}\;\mathrm{to}\;\mathrm{discharge}\;\mathrm{saliva} \end{array}$$

## 3. Experiments

### 3.1. Participants

This study was conducted in accordance with the amended declaration of Helsinki. The Hiroshima Cosmopolitan University Institutional Review Board (No. 2015031) approved the protocol, and written informed consent was obtained from all participants. We performed experiments on 33 young participants (21.3 ± 0.4 years) and 25 elderly participants (80.4 ± 6.1 years) (see Table 1). The participants consisted of self-reported healthy individuals with no previous cardiovascular or pulmonary diseases. Before the cough sound measurements, we measured the %VC, FEV_1_, and FVC, and excluded those participants with FEV_1_/FVC < 80% and those who could not perform the measurement. As a result, two elderly participants with FEV_1_/FVC < 80% were excluded, representing 3.3% of all participants.

### 3.2. Methods

#### 3.2.1. Cough Flow and Sound Measurement Methods

To measure cough flows, the participants wore a face mask with a flow sensor (Autospiro AS-507; Minato Medical Science Co., Ltd., Osaka, Japan) attached and performed coughing three times in a sitting position. The measurement range of the flow sensor was 0–840 L/min, and the measurement accuracy was within 3% of the indicated value. Hereafter, the measured CPF refers to the cough peak flow calculated from the maximal value obtained from the flow sensor.

To measure cough sounds, the smartphone microphone was used. As explained in Section 2.2, the *CPSL* was calculated to estimate the CPF. The proposed model was then compared with other possible equation forms to analyse the effects of age, height, weight, and BMI on the estimation accuracy. It should be noted that the sex of the participants had a minimal effect on the CPF estimation accuracy, as we have previously reported [7,8]. Hereafter, we denote the CPF estimated by the proposed model as *CPS_proposed_* and those estimated by the model shown in Equations (2)–(6) explained in the following subsections as *CPS_X_*, where the subscript *X* distinguishes the model used.

#### 3.2.2. Models for Age Effect Analysis

We hypothesized that the *CPSL* is proportionally affected by age based on a previous study [9]. To validate this hypothesis, we compared the estimation accuracy with that of our previous model, expressed by Equation (2), in which the age effect is not included [7,8]. In addition, the proposed model was compared with the models that include the second- (Equation (3)) and third-order (Equation (4)) terms of the *age* variable, as follows:

(2) $$CPS_{previous}=\alpha \cdot \left(exp^{\beta \cdot CPSL}-1\right),$$

(3) $$CPS_{age2}=\left(\alpha_{0,\left(3\right)}+\alpha_{1,\left(3\right)}\cdot age+\alpha_{2,\left(3\right)}\cdot age^{2}\right)\cdot \left(exp^{\beta_{\left(3\right)}\cdot CPSL}-1\right),$$

(4) $$CPS_{age3}=\left(\alpha_{0,\left(4\right)}+\alpha_{1,\left(4\right)}\cdot age+\alpha_{2,\left(4\right)}\cdot age^{2}+\alpha_{3,\left(4\right)}\cdot age^{3}\right)\cdot \left(exp^{\beta_{\left(4\right)}\cdot CPSL}-1\right),$$

where *α*_0,(*Y*)_ to *α*_3,(*Y*)_ and *β*_(*Y*)_ are constant parameters determined by the Levenberg-Marquardt method using the mean square error between the *CPS*_X_ and measured CPF as the evaluation function; the subscript, *Y*, distinguishes the model. The 95% CIs are also calculated for each parameter. In addition, the parameters included in Equation (2) are determined by calculating the CPS using a previous model, such as *α* = 70.98 and *β* = 0.022, based on our previous study [8]. The Wilcoxon signed-rank test was used to compare the absolute error between the previous and proposed models. Spearman’s rank correlation coefficient analysis was used to assess the relationship between each *CPS*_X_ and measured CPF. Absolute reliability was investigated using the Bland-Altman analysis method to detect systematic bias, such as fixed and proportional bias.

#### 3.2.3. Analysis of Models for Body Weight, BMI, and Height

We verified the effects of body weight, BMI, and height on the prediction accuracy because a previous study reported that the CPF can depend on body weight and height [9]. In this study, we hypothesized that the body weight and/or BMI proportionally increases with increasing CPF, and that the height proportionally decreases with increasing *CPSL* because taller participants have longer arms and the increased distance between the smartphone microphone held in the hands (Figure 1a) and the mouth attenuates the sound level. We included the terms for body weight, BMI, and height, as follows:

(5) $$CPS_{wB}=\left(\alpha_{0,\left(1\right)}+\alpha_{1,\left(1\right)}\cdot age+\alpha_{w}\cdot weight+a_{B}\cdot BMI\right)\cdot \left(exp^{\beta_{\left(1\right)}\cdot CPSL}-1\right),$$

(6) $$CPS_{height}=\left(\alpha_{0,\left(1\right)}+\alpha_{1,\left(1\right)}\cdot age\right)\cdot \left[exp^{\beta_{\left(1\right)}\cdot \left\{CPSL+20\log\left(height/d_{0}\right)\right\}}-1\right],$$

where *α*_w_, *α*_B_, and *d*_0_ are constant parameters determined using the same method described in the previous section; *weight*, *BMI*, and *height* represent the participant’s body weight, BMI, and height, respectively; and *α*_0,(1)_, *α*_1,(1)_, and *β*_(1)_ are the same values as those determined for the proposed model. The second term in the exponential function of Equation (6) (the height model) represents the correction term for the attenuation related to the participant’s height. A decrease in the sound level, *L_p_*, can be calculated by the distance (*r*, *r*_0_) between the sound source and the microphone, as follows:

(7) $$L_{p}=20\log\left(r/r_{0}\right),$$

where *r*_0_ and *r* are constants. Thus, to correct the *CPSL* for additional sound attenuation, *height* was inserted in Equation (6) instead of *r*. In this analysis, the coefficient of determination and the 95% CI were calculated for comparison with the proposed model (Equation (1)).

All statistical tests in this paper assumed a significance level of 0.05, and analyses were performed using G*power (version 3.1.9.2; University Kiel, Kiel, Germany) and IBM SPSS Statistics 24.0 (IBM, Chicago, IL, USA).

## 4. Results

### 4.1. Parameter Determination

Table 2 shows the determined parameters. In the proposed model (Equation (1)), the coefficients *α*_0_, *α*_1_, and *β* are as follows: *a*_0_ = 42.90 (95% CI: 7.84 to 77.96), *a*_1_ = −0.282 (95% CI: −0.509 to −0.055), and *β* = 0.028 (95% CI: 0.020 to 0.037); the determination coefficient of the proposed model is 0.829. In Equations (3) and (4), the coefficients are determined in the same manner as in the proposed model. Equation (3) yielded a determination coefficient of 0.829. The determined parameters are as follows: *a*_0__,__(3)_ = 42.32 (95% CI: 7.15 to 77.50), *a*_1__,__(3)_ = −0.212 (95% CI: −0.713 to 0.289), *a*_2__,__(3)_ = −0.001 (95% CI: −0.006 to 0.004), and *β*_(3)_ = 0.028 (95% CI, 0.020 to 0.037). The 95% CIs of the coefficients, *a*_1,(3)_ and *a*_2,(3)_, in Equation (3) include 0. Equation (4) yielded a determination coefficient of 0.832. The determined parameters are as follows: *a*_0__,__(4)_ = 6.58 (95% CI: −70.33 to 83.49), *a*_1__,__(4)_ = 2.366 (95% CI: −3.448 to 8.181), *a*_2__,__(4)_ = 0.00 (95% CI: 0.00 to 0.001), *a*_3__,__(4)_ = −0.048 (95% CI: −0.156 to 0.060), and *β*_(4)_ = 0.028 (95% CI, 0.020 to 0.037). The 95% CIs of all parameters, except for *β*_(4)_ in Equation (4), include 0. The 95% CI indicates that only *α*_0,Y_, *α*_1,Y_, and *β*_Y_ are valid parameters because the 95% CIs of the other parameters include 0.

### 4.2. Estimation Accuracy

**Figure 4:**
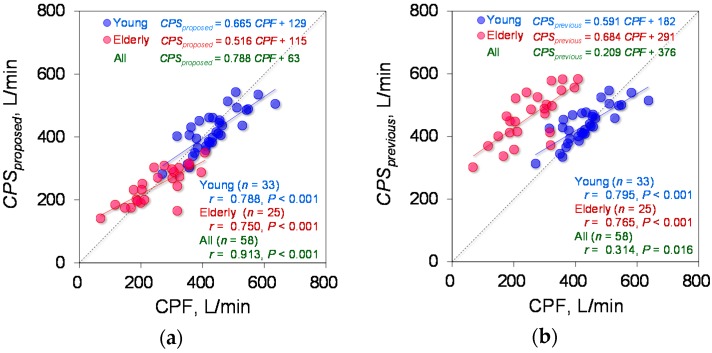
Estimation accuracy of *CPS_proposed_* and *CPS_previous_*. (**a**) The CPF estimated by the proposed model (*CPS_proposed_*) against the measured CPF. (**b**) Plot of the CPF estimated by the previous model (*CPS_previous_*) against the measured CPF. The red and blue circles represent the elderly and young participants, respectively. The linear regression lines are drawn for the groups of young and elderly participants, and the corresponding equations are shown in the upper part of each figure, where the green letters indicate the equation of the regression line for all participants. The right lower side shows the correlation coefficients and *P* values for each participant group.

**Figure 5:**
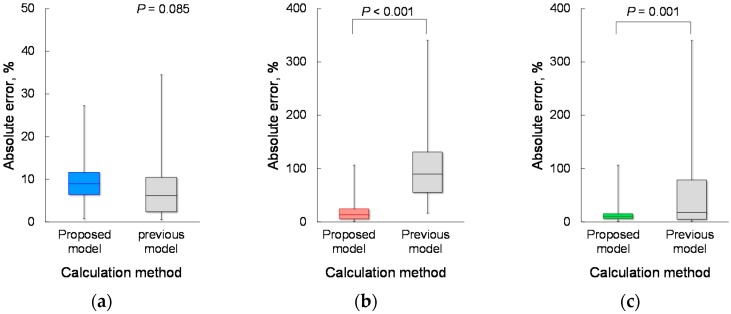
Comparison of the absolute error between the proposed and previous models. (**a**) Young participants, *n* = 33. (**b**) Elderly participants, *n* = 25. (**c**) All participants, *n* = 58.

**Figure 6:**
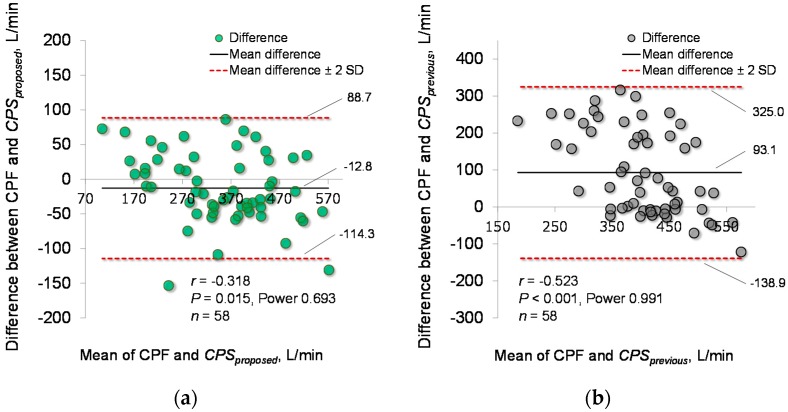
Bland-Altman plots of the measured and estimated CPFs. (**a**) The estimation accuracy of the proposed model (*CPS_proposed_*). (**b**) The estimation accuracy of the previous model (*CPS_previous_*). The horizontal line is the mean of the measured CPF and estimated cough peak flow (*CPS_X_*). The vertical line represents the difference between the measured CPF and *CPS_X_*. The bold black solid lines represent the mean differences between the CPF and *CPS_X_*, and the red dotted lines represent the mean differences ± 2 standard deviations.

Based on the determined parameters, the CPF estimation accuracy of the proposed model (Equation (1)) was verified. Figure 4a demonstrates the relationship between each *CPS_proposed_* and measured CPF in the young and elderly participants. To compare the CPF estimation accuracy of the proposed and previous models, Figure 4b shows a plot of the *CPS_previous_* against the measured CPF. Spearman’s rank correlation coefficient analysis showed a significant positive correlation between the *CPS_proposed_* and measured CPF in both the young participants (*r* = 0.780; *p* < 0.001; power > 0.99) and the elderly participants (*r* = 0.750; *p* < 0.001; power > 0.99). For all participants, the Spearman’s rank correlation coefficient is *r* = 0.913, with *p* < 0.001 and power > 0.99, as shown in Figure 4a. In addition, Spearman’s rank correlation coefficient analysis showed a significant positive correlation between the *CPS_previous_* and CPF (young participants: *r* = 0.795; *p* < 0.001; power > 0.99, elderly participants: *r* = 0.765; *p* < 0.001; power > 0.99, all participants: *r* = 0.314; *p* = 0.016; power, 0.68), as shown in Figure 4b. Moreover, in young participants, the Wilcoxon signed-rank test showed no significant differences in the absolute error between the *CPS_proposed_* and *CPS_previous_* (6.19% vs. 8.95%, *p* = 0.085) (Figure 5a); however, in the elderly participants and all the participants, the Wilcoxon signed-rank test showed significant differences in the absolute error between the *CPS_proposed_* and *CPS_previous_* (13.55% vs. 90.01%; *p* < 0.001, 9.96% vs. 17.92%; *p* = 0.001, respectively) (Figure 5b,c). In addition, Figure 6 shows the corresponding Bland-Altman plots for the proposed and previous models. Neither model showed fixed bias, but both models showed proportional bias (*r* = −0.318; *p* = 0.015; power, 0.693, *r* = −0.523; *p* < 0.001; power, 0.991, respectively).

### 4.3. Effects of Body Weight and BMI on CPF Estimation Accuracy

To consider the effects of body weight and BMI on the CPF estimation accuracy, Equation (5), which includes terms for body weight and BMI, was tested. The parameters, *a*_w_, *a*_B_, and *d*_0_, were determined as explained in Section 3.2.2. The determined parameters are as follows: *a*_w_ = 0.137 (95% CI: −0.021 to 0.295) and *a*_B_ = −0.303 (95% CI: −0.717 to 0.110). The 95% CIs of coefficients, *a*_w_ and *a*_B_, in Equation (5) include 0, indicating that these parameters are not valid. Equation (5) yielded a determination coefficient of 0.839.

### 4.4. Effect of Body Height on CPF Estimation Accuracy

**Figure 7:**
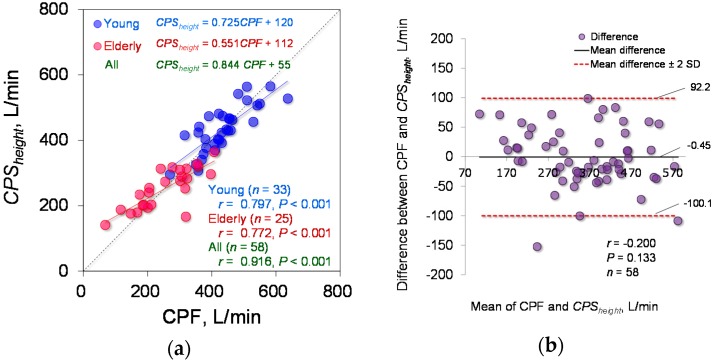
Estimation accuracy of *CPS_height_*. (**a**) *CPS_height_* against the measured CPF. The linear regression lines are drawn for the young and elderly participant groups, and the corresponding equations are shown in the upper part of each figure, where the green indicates the equation of the regression line for all participants. The right lower part shows the correlation coefficients and *p* values for each participant group. (**b**) Bland-Altman plot of the measured CPF and *CPS_height_*. The horizontal line is the mean of the measured CPF and *CPS_height_*. The vertical line represents the difference between the measured CPF and *CPS_height_*_._ The bold black solid lines represent the mean difference between the measured CPF and each *CPS_height_*, and the red dotted lines represent the mean difference ± 2 standard deviation bands.

The estimation accuracy of Equation (6), which includes a term for height in the exponential function, was tested. The determined parameter is *d*_0_ = 141.6 (95% CI: 122.8 to 160.5). Equation (6) yielded a determination coefficient of 0.833. Spearman’s rank correlation coefficient analysis showed a significant positive correlation between the *CPS_height_* and measured CPF (young participants: *r* = 0.797; *p* < 0.001; power > 0.99, elderly participants: *r* = 0.772; *p* < 0.001; power > 0.99, all participants: *r* = 0.916; *p* < 0.001; power, 1), as shown in Figure 7a. In addition, Figure 7b shows the corresponding Bland-Altman plot of Equation (6). Equation (6) did not show fixed or proportional bias (*r* = −0.200; *p* = 0.133).

**Figure 8:**
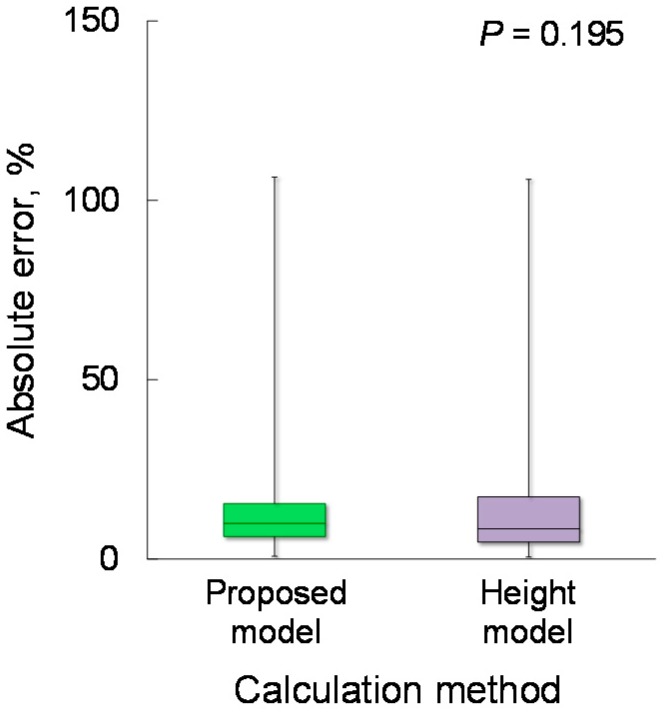
Comparison of the absolute error of the proposed and height models. *n* = 58.

To compare the estimation accuracy of *CPS_proposed_* and *CPS_height_*, the absolute error was calculated. The Wilcoxon signed-rank test showed no significant differences in the absolute error between *CPS_proposed_* and *CPS_height_* (9.96% vs. 8.44%; *p* = 0.195), as shown in Figure 8.

### 4.5. Examples of Elderly Participants

**Figure 9:**
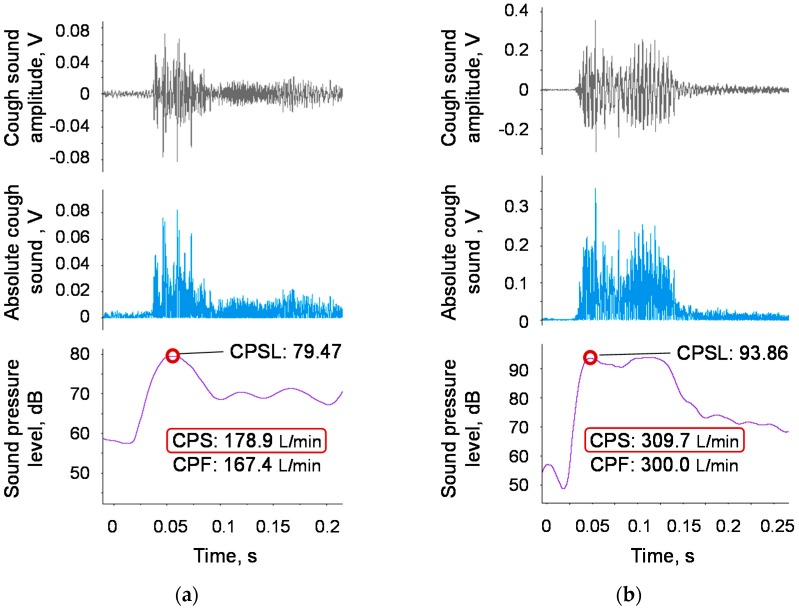
Examples of elderly participants. The grey line represents the measured cough sound signal. The light blue solid line represents the bandpass-filtered and rectified sound signal. The purple solid line represents the moving average with a time window of 20 ms. The red circle represents the maximum point of the moving average. (**a**) Example of a 77-year-old female with a measured CPF below the reference value of 270 L/min. The respiratory function test showed slightly low values of %VC = 83.5% and FEV_1_/FVC = 83.0%, but these values exceed the reference value. (**b**) Example of a 70-year-old male with a measured CPF above the reference value of 270 L/min. The respiratory function test showed normal values of %VC = 91.6% and FEV_1_/FVC = 94.9%.

Finally, we performed experiments using the proposed device, implementing the proposed model (Equation (1)). Figure 9a,b shows examples of two elderly participants in which the measured CPFs were below and above the reference value of 270 L/min.

## 5. Discussion

Aiming to establish a method for evaluating cough ability that can be applied in daily clinical practice, we propose a model that can convert cough sounds into cough peak flow (CPF) values, and we developed a user interface for mobile devices, such as a smartphone, that makes it easy for both patients and caregivers to use the proposed method. To the best of our knowledge, this is the first study to clarify that CPF estimation using cough sounds requires a model involving the age factor. In addition, we found that including the height factor can slightly improve the estimation accuracy.

We hypothesized that age affects the estimation accuracy of the CPF based on the fact that a previous study reported a relationship between the CPF and age [9]. Analysis of the age factor revealed that the proposed model, which includes a first-order term for age (see Equation (1)), is sufficient to estimate the CPF from cough sounds (see Figure 4a) and that second- or higher-order terms for age (see Equations (3) and (4)) can be ignored because the 95% CI of the determined parameters included 0 (see Table 2). A comparison of the absolute error between the proposed and previous models [7,8] (Equation (2)) showed a significantly improved estimation accuracy among elderly participants for the proposed model (see Figure 4). This finding indicates that age has a major effect on the CPF estimation accuracy. The age factor may also reflect the relationship between the cough strength, vital capacity [12,13,14], and vocal fold function [15,16,17,18,19].

A previous study reported that the CPF depends on body weight and height [9]. Based on this previous study, we tested a model including terms for body weight and BMI (see Equation (5)); however, the 95% CI of the determined parameters of these terms (*α*_w_ and *α*_B_) included 0 (see Section 4.3), indicating that *weight* and *BMI* have minimal effects on the CPF estimation accuracy.

A previous study also reported that the distance between the mouth, which is the sound source, and the microphone needs to be less than 30 cm because the recorded cough sounds attenuate with increasing distance from the sound source [7]. In addition, recoded sounds are affected by reflection from walls because sound propagation in a room is a combination of direct and reflected sound waves from surfaces and boundaries in the room [20]. In the context of the daily applicability of the proposed method, the mobile device was handheld (see Figure 1a), and we did not precisely specify the distance between the mouth and the microphone. As a result, the median distance between the participant’s mouth and the smartphone microphone was approximately 37.50 cm, and the interquartile range was approximately 6.25 cm. This indicates that recorded cough sounds can be influenced by sound reflection and attenuation, and that the distance varied among the participants. This could be one reason the Bland-Altman plot of the proposed model (see Figure 6a) showed proportional bias. One possible solution is to attach the microphone to the face to maintain a constant distance between the microphone and the sound source [7]. In this study, another solution was tested, in which the height term was included in the CPF estimation model to compensate for the attenuation (see Equation (6)) because there was a significant positive linear relationship between the participant’s height and the distance between the sound source (participant’s mouth) and the microphone (*r* = 0.688; *p* < 0.001; power > 0.99). We found that this model successfully eliminated the proportional bias (see Figure 7b). However, a comparison of the absolute error between the proposed model and the model including the height term showed no significant difference. Considering daily use, we adopted the model proposed in this paper, which does not require measuring the participant’s height. However, there is a possibility that the model including the height term is more suitable for different ethnic groups in which height varies greatly, as the participants in this study were Japanese and their height did not drastically vary (see Table 1).

A major limitation of this study is that we did not fully consider cough sound frequencies, although previous studies have suggested that breath sounds can be influenced by sound frequencies [21,22]. The estimation accuracy could thus be further improved by considering the frequency domain. In addition, because the proposed method was aimed at the daily evaluation of cough ability and risk screening, it is implicitly assumed to be applicable to healthy or close to healthy users. However, we are also expecting a person with a disease or airway mucous to use the proposed method. In this regard, we did not fully consider the effects of disease and airway mucous, since the participants were healthy volunteers. It is thus necessary to verify the proposed method in patients for further clinical application.

## 6. Conclusions

This paper presents a cough strength evaluation based on cough sounds considering the effect of age and designed for daily use in clinical practice along with a custom-designed user interface. The experimental results confirm that the age term improves the CPF estimation accuracy and that height can also affect the estimation accuracy. This study found that body weight and BMI have minimal effects on the CPF estimation accuracy. This is an unexpected result because previous studies [9] reported CPF correlates with body weight. This indicates that cough sound may carry information about body weight and allows CPF estimation without using weight as a parameter; this is a novel fact revealed by this study. The experiment results also revealed that cough strength can be evaluated in elderly people by using the proposed model and device. This finding indicates that the sound quality may change with age, but its effect on CPF estimation can be compensated by adding a proportional age coefficient; this is another novel finding of this study. Toward practical application, we plan to test the efficacy of the proposed model and the user interface software implemented on a mobile device for daily use during in-home care.

## Figures and Tables

**Table 1:** Characteristics of the participants.

Variable	Young Participants (*n* = 33)	Elderly Participants (*n* = 25)	*p* Value
Age, years	21.3 ± 0.4	80.4 ± 6.1	< 0.001
Male sex, *n*	20	10	0.209
Height, cm	164.6 ± 8.4	154.1 ± 8.3	0.093
Body weight, kg (male, female)	58.5 ± 11.6 (64.5 ± 11.3, 51.0 ± 6.2)	55.1 ± 11.9 (58.6 ± 6.3, 53.4 ± 15.1)	0.156
BMI, kg/m^2^ (male, female)	21.3 ± 0.5 (21.4 ± 0.6, 20.9 ± 0.5)	23.3 ± 4.1 (22.5 ± 1.6, 24.0 ± 5.2)	0.216
%VC, %	97.0 ± 8.9	91.5 ± 17.5	0.438
FEV_1_/FVC, %	90.4 ± 7.6	91.8 ± 8.2	0.185

Values presented are the number of participants or the mean ± S.D. of the corresponding parameters, unless otherwise stated. BMI, body mass index; VC, vital capacity; %VC, vital capacity expressed as a percentage of the predicted value; FEV_1_, forced expiratory volume in 1 s; FVC, forced vital capacity.

**Table 2:** Determined parameters.

Model	Parameter	Determined Value	Standard Error	95% CI		Determination Coefficient
				Lower	Upper	
Equation (1)	*α*_0,(1)_	42.90	17.50	7.84	77.96	0.829
	*α*_1,(1)_	−0.282	0.113	−0.509	−0.055	
	*β*_(1)_	0.028	0.004	0.020	0.037	
Equation (3)	*α*_0,(3)_	42.32	17.54	7.15	77.50	0.829
	*α*_1,(3)_	−0.212	0.250	−0.713	0.289	
	*α*_2,(3)_	−0.001	0.002	−0.006	0.004	
	*β*_(3)_	0.028	0.004	0.020	0.037	
Equation (4)	*α*_0,(4)_	6.58	38.34	−70.33	83.49	0.832
	*α*_1,(4)_	2.366	2.899	−3.448	8.181	
	*α*_2,(4)_	0.000	0.00	0.00	0.001	
	*α*_3,(4)_	−0.048	0.054	−0.156	0.060	
	*β*_(4)_	0.028	0.004	0.020	0.037	
